# Is There a Need for a More Expansive Use of Ethics and Values in Reflecting on the Use of Animals in Scientific Research?

**DOI:** 10.3390/ani4040643

**Published:** 2014-10-10

**Authors:** Mark W. Fisher

**Affiliations:** Kotare Bioethics, Wellington, New Zealand; E-Mail: kotare.bioethics@xtra.co.nz

**Keywords:** animal research, benefits, harms, animal rights, ethics, respect, care, virtues, experiences

## Abstract

**Simple Summary:**

Debates about animal research are often polarized. On one side, increased knowledge, medical treatments or enhanced animal production are seen to justify harms. On the other, animals have rights constraining their use. Both sides fail to provide adequate support for this contentious use of animals. Reflecting the richness of ethics, it is suggested that empathy and respect for the animal, and greater acknowledgement of the value of experiences and care could also be considered. Along with a more informed understanding of the benefits of research and of the aims of animal rights, a more sophisticated ethic may reflect the common ground between the poles of the debate.

**Abstract:**

Although reflecting a long tradition of moral reflection that the use of animals is acceptable as long as it is humane, the tension between causing harm to animals in research and the benefits to humans can nevertheless be troubling. Utilitarian arguments that appeal to the value of those practices in sustaining and enhancing human lives, and rights-based arguments which seek to constrain them, can be inadequate. Reflecting a more engaging, inclusive and sophisticated understanding of human activity, justification for animal use could be expanded to reflect the fullness and richness of ethical thinking. This might see more explicit inclusion of perspectives borne of virtues, caring, experiences, and respect for the essence of the animal, and different ways of understanding and knowing animals, values drawn from the middle ground of commonly acceptable human-animal relationships. Such values, already clearly evident in research, could be more widely integrated into arguments justifying animal use. A more expansive approach would not only reflect reality and acknowledge that costs and benefits are shared more widely, but it might result in more equitable, effective and humane science. It might also serve to reduce some of the tension long evident in the relationship between humans and animals.

## 1. Introduction

*I was so lucky. I had a horse that was half-human and a trainer that was half-horse. What a combination—*Keith Haub, part-owner of McGinty [1]. That quotation, taken from the thoroughbred racing industry, is a reminder of how important and complex, and inextricably linked, are the relationships between animals and humans. Animals are sources of labor, food, clothing, accessories, wealth, learning, entertainment, companionship, competition and cultural symbols. The foundation for many of these relationships was an “ancient contract” [2]. “We take care of the animals and the animals take care of us” reflects the co-evolution of people and domestic animals. The “contract” has long been a source of tension and is consequently accompanied by the belief that since we are indebted to them we have a duty to consider animals’ interests [3,4,5]. Animals in scientific research, testing and teaching are no different. In an early account, from classical Rome, Galen, a court physician, dissected apes and pigs to understand the body, while his Emperor, Marcus Aurelius, believed that we should “treat dumb animals and generally all things and objects with generosity and decency” [6,7].

In sustaining and enhancing our lives, the interests of humans are promoted at a cost to animals and the tension evident in Roman days persists. On the one hand, “many who read this would not be alive and healthy today if not for animal and human experimentation so the question is not whether one believes in experimenting with animals but what is the nature of the experiment” [8]. Furthermore, since “history teaches us that we cannot know what we need to know until we know what there is to know” [9] it would seem much experimentation is justified. On the other hand, it is also held that “harms of a certain degree and kind ought under no circumstances to be inflicted upon an animal” regardless of any benefits [3]. Similarly, “there is no reason to do as much of the harmful research as is done these days” [10]. The tension is most visible, at least publicly, in the passion, beliefs and lengths, unfortunately in some cases criminal, to which some people go to protest against, or to support, the use of animals in science [11,12,13,14].

How are these opposing views reconciled? The use of animals in science, and compromises to their welfare, is commonly characterized by an appeal to the benefits of using them, and a general “dismissal” of animal rights. Reasonable people can disagree and those disagreements need to be discussed—for those who find animal research problematic, even troubling, such simplistic and often entrenched stances, even dismissal, are inadequate, failing to reflect the fullness and richness of human abilities to reflect upon and deal with complex issues. The real value of ethics in providing different insights into complex issues appears to have been overlooked. In this personal reflection based on several decades of involvement in science and ethics (e.g., [15,16,17]) it is suggested that the debate over the use of animals in science could benefit from a more engaging, inclusive or sophisticated understanding of human activity—that actions or conduct involves making choices with certain consequences, within the constraints imposed by society [18]. Aspects of emphasis on the benefits of animal research and dismissals of advocacy for animal rights are discussed, followed by a middle ground stance based on virtues and other values, a reflection of the ancient contract. If these views are sound, they raise a number of opportunities for better understanding and justifying the use of animals in scientific research.

## 2. Difficulties with Common Ethical Stances

The predominant justification for the use of animals in research, testing and teaching requires good reason to believe the benefits (to humans, animals or ecosystems) derived from the use of animals must outweigh the likely harms to those animals. For example, bloat is caused by the accumulation of gas in the rumen, primarily when cattle feed on legumes or lush young grass. It may result in distension of the abdomen, restlessness, straining and discomfort, difficulty in breathing, and in severe cases death [19]. Animal experimentation [20,21] involving manipulations such as the surgical placement of a fistula in the wall of the rumen to enable sampling of its contents, led to the control of bloat with anti-foaming agents, a technology used to control bloat in the dairy industry. Any fear, discomfort, pain or distress unavoidably caused to experimental animals is typically justified by the benefits of understanding bloat and of developing methods for preventing and treating it.

Despite being contentious human activities can have benefits. For example, the courage and leadership shown by people during times of war (e.g., Charles Upham, Winston Churchill) is highly valued. Similarly, during the Second World War, the United Kingdom set up a “Digging for Victory” campaign to ensure the health of the military, mothers and children. Providing food of good nutritional quality (vegetables and milk) through, among other things, turning parks into vegetable gardens, had the additional benefit of a fall in deaths from heart disease [22]. Or the microwave oven, a descendant of the development of radar, also in the Second World War [23]. While acknowledging these benefits, it is accepted that they do not justify war itself. Although a limited and simple analogical argument, it suggests that relying exclusively on an ethic based on the benefits outweighing the harms is problematic. Furthermore, justifying animal research by presenting “a rich bouquet of carefully picked examples” commits the fallacy of selective perception, counting successes and ignoring failures [24]. Additionally, animal research usually involves a complex set of compromises or insults to animals, some of which are not necessarily related to the likely benefits. For example, animals are often housed in confined and barren environments for methodological, practical and economic reasons rather than for the outcomes of the experiment. These reasons may be good (e.g., standardization of experimental conditions reduces variability increasing precision and reproducibility though limiting the wider applicability of the results [25,26]) but is it sound to justify them by appealing to the benefits of experimentation? If the animals were kept in environments more in keeping with their needs or natures, would not the compromises associated with experimentation be more easily justified?

The second difficulty noted with justifying animal research is the apparent dismissal of animal rights perspectives, both by advocates of animal research and, seemingly, by legislation [27]. One view is that animals are the subject of a life and therefore have a right not to be used in animal research, as well as be farmed or kept as pets [28]. While there is likely wide empathy with some of these positions, e.g., objecting to killing animals for the production of a luxury such as fur [29], a simple analysis reveals difficulties (e.g., [30]). Among them is the belief that humans cannot live without having some impact on animals [31], arguably more so if all humans were vegetarians [32,33]. If, as has been suggested [34,35], domestic animals and humans have co-evolved then have not humans a responsibility for at least some sort of relationship with animals? Few people see the world as exclusively human but acknowledge humans form compassionate and caring bonds with other creatures [36]. Man does not naturally live in species isolation but it is a peculiar human talent to draw in, domesticate and live with, rather than ignore, a great variety of other species—no other animal does so on such a similar scale. The animal rights view based on subjects of a life has been described by its author as revolutionary and perhaps it is valued more for its novel philosophical engagement than in providing practical guidance of the human-animal relationship. While it is understandable, then, that animal rights is often dismissed, a synonym for views outside the norm, especially when associated with illegal activities, such a stance does not reflect the complexity and common understanding of the term, nor its potential to contribute to a shared ethic.

Rights are a social device that makes it easier for people to live with each other by providing a protection or constraint on treatment and animal rights theory has a long history (e.g., see Salt, 1894 [37]). The issue for many people is not really about the view of rights described above, but something more moderate. Animal rights (e.g., “a right that no useless pain or misery be inflicted”) appear to have evolved from 18th century reaction to humans apparently having no obligation to animals or to their treatment. Not surprisingly, the movement resulted in almost complete consensus in the need for speedy killing of animals when slaughtering or in eradicating vermin, and in repudiating cruelty to animals [38]. This view of rights is reinforced by the idea that animal welfare and animal rights are, despite common perceptions, essentially similar in aims [39,40]—animals have entitlements or rights to adequate food and shelter *etc*., which humans have a duty to provide (both for the animals sake and because it makes us better human beings). It is a view seemingly widely shared—75% of respondents in one survey agreed that animals have rights that people should respect [41]. “Animal rights” can, and commonly does, refer to any call for the fair treatment and protection of animals.

A more sophisticated understanding of rights, especially one engaging concepts and values such as animal integrity, *telos*, reverence, sympathy, respect, wonder and awe—a life worth living—is likely to contribute to the future of the animal-human relationship [42,43,44,45,46,47]. However, it will require society to, for example, understand and agree to the particular nuances of philosophical, legal, cultural and political insights, and ensure they are grounded in biology, as ethics demands [48]. The challenge is to present these concepts in ways which are easily understood and support progress to a more equitable and sustainable future, both for animals and humans. This has frequently not been the case, no doubt contributing to the general dismissal of animal rights. Furthermore, as has been suggested, animal scientists and animal rights activists have many shared goals (as well as simplistic and stereotyped views of each other). Combining their respective strengths of knowledge and skills, and motivation, organization and political influence may well result in mutual cooperation [49,50,51,52].

Following this approach, then, it is suggested that appeals to rights by opponents of animal research, or to benefits, by advocates of animal research are inadequate. Whilst acknowledging the importance of polarized positions in helping define the issues, it is the middle ground between the poles of opposition and advocacy [52,53,54] which perhaps provides the more insightful opportunities for understanding and guiding human-animal relationships. If the middle ground between advocacy and opposition to animal research is where the relationship between animals and man flourishes, in the true spirit of co-evolution, then it is suggested that that is where the ethics of animal experimentation should be drawn from and debated. In fact, this ground may reflect an innate sense of intuitive ethics [55] before the reductionist-like descriptions of its components. Furthermore, and arguably most importantly, the middle ground is where change may be constructively considered [56] to improve animal welfare and realistically discuss the implications of various ethical insights e.g., animal rights. In the next section, science and the humanities are used to illustrate different human understandings of animals, understandings, it is suggested, that lie between relying solely on the benefits of animal research to justify it or of animal rights to prohibit it.

## 3. Knowing Animals

A major principle defining our relationship with animals, and one which reflects a long tradition of moral reflection, is that the use of animals is acceptable providing that that use is humane [3]. The ancient context was based on husbandry—a respect for the essence of the animal—a duty of care. Care begins with knowing, especially of knowing the animal. It is a central tenet of good stockmanship, which is also inextricably linked with the character, or nature of the stockman [57,58].

Therefore, how can we know animals, the rat, for example? There are many perspectives including that of science. Burn [59] summarized eight decades of scientific research on how rats see their world, with the following:

*The rat’s sensitive eyes, shunning the intense artificial light, provide it with a hazy view in predominantly grey, ultraviolet and green hues. From within its cage, it hears the chirps, squeaks and whines of its neighbors, gaining information that we cannot hear unaided and are yet to understand. Background noise consists of the low babbles and hisses of distinctively scented humans, and the unregulated drones and blasts of ultrasonic sounds. Scents provide visceral warnings and enticements, induce new motivations, and inform the rat about social possibilities outside the cage. The environment wafts a succession of scents, from pleasant, calming fragrances to the innately alarming odours of intangible predators. The rat tastes little apart from its dry, satiating homogenous diet. Its vibrissae provide a protective, finely tuned force field to feel the details of the cage surfaces; with the rat perceiving security from close contact with the solid walls*.

Natural history also provides a perspective. For example, the documentary *The Laboratory Rat: A Natural History* [60] follows the lives of domestic rats released into an outdoor enclosure. In their new environment, the rats have to compete for food, shelter and mates and the film shows the emergence of a complex and structured society, which soon thrives in the wild environment.

Stories, especially those making use of individuals’ experiences, can help us to imagine animals’ worlds. *The War of the Worlds* [61] invites understanding of a rat’s plight:

*Strange night! Strangest in this, that so soon as dawn had come, I, who had talked with God, crept out of the house like a rat leaving its hiding-place—a creature scarcely larger, an inferior animal, a thing that for any passing whim of our masters might be hunted and killed. Perhaps they also prayed confidently to God. Surely, if we have learnt nothing else, this war has taught us pity—pity for those witless souls that suffer our dominion*.

Anthropomorphism, attributing human traits to animals, is a sometimes neglected even “ostracized” method of understanding animals [62,63]. The novel *Firmin* [64] tells of a rat that lives above a bookstore; the rat’s world more like our own:

*My favorites were the Modern Library editions ... At first I just ate happily gnawing and chewing ... When Norman made an especially big sale … I clapped my paws and silently shouted, “Way to go, Norm!”... He had seen me! … a little heap of strange food. A little pile of cylindrical neon green pellets. They smelled good, so I nibbled. … I woke up coughing and full of anguish. I tried to vomit but couldn’t*.

*The Limits of Trooghaft* [65] imagines what it is like to be exploited as animals by aliens—“The Troogs took one century to master the planet, then another three to restock it with men, its once dominant but now conquered species” now segregated as housemen (pets), capons (battery-reared sources of food), swift ferocious houndmen (hunters) and quarry-men (nomadic rebels, wild or natural humans).

Finally, and arguably most importantly, we can know animals by having an empathy with them [66]:

*There is another truth that I would not deny for a second namely that those rare humans who have a real gift for training and working with dogs owe that gift to experience, intuition and a certain kind of empathetic reasoning that has almost nothing to do with science*.

Empathy, one of the qualities of stockmanship, is based on a respect for the essence of the animal [57,58] part of the human-animal bond [5]. Furthermore, it is perhaps people seemingly with skills for living in right relationships between humans and the natural world, relationships borne of respect, which society should be drawing on [67].

Many people “know” animals in different ways including animal technicians who arguably know much more of research animals’ worlds than many other people. For instance, animal technicians are very good at detecting post-operative clinical problems such as wound infections, dehydration, and self-mutilation—perhaps 90% are detected compared with only 10% by researchers [68]. Similarly, subtle changes, such as a reversal of dominance hierarchies in rabbits associated with induced cystitis were missed by a veterinarian but observed by a technician [69]. Such observations resemble those in farming—the first indication of an illness in housed sheep was the animal not looking the stockman in the eye [70]. The defining characteristic of these people is, perhaps in addition to their empathy, their broad understanding, that they know the norms so that they can see the abnormal; they have some sort of innate ability or animal sense akin to stockmanship, a respect for the essence of the animal [58]. It is suggested that traditional ethical reflection on the use of animals in science makes little explicit use of this knowledge of animals, consequently risking a relationship based on minimal respect for animals.

## 4. Respect for the Research Animal

Empathy for, and attention to animals’ needs is not only a central tenet of good animal welfare, but also reflects a respect for the animals’ nature [71]. Caring is part of human nature but it is suggested that animal science sometimes gives less than full acknowledgement to the skills and experiences of those actually interacting with and caring for animals on a regular basis, be they animal technicians or farm staff. For instance, the well-known principles of humane experimental technique—replacement, refinement and reduction or the Three Rs [72]—are generally directed at researchers or scientists and reinforced at conferences and in scientific publications (e.g., [73]). While not doubting the importance of those initiatives, is the knowledge of animal technicians and farm staff being overlooked in promoting the Three Rs? Animal staff that place plastic containers in cages of rats with diabetes insipidus so that they have access to dry areas, are probably motivated more by an intuitive or innate sense of care than by explicit knowledge of the Three Rs. It is suggested that animal science could better acknowledge and incorporate the knowledge, borne of experience and empathy, of those routinely interacting with and caring for experimental animals. There are many ethical theories which could contribute to a more engaging ethic justifying the use of animals in research [74,75]. For example, an ethic of care emphasizes a commitment and willingness to act on behalf of those with whom we have a relationship while virtue ethics is founded on the strengths of character that promote a good life. Perhaps even casuistry or case-based reasoning which depends on an intimate understanding of the particular situation in a historical context. Ethical theories drawing on experiences, feelings and emotions, e.g., respect and compassion, may complement the more dominant justification based on the benefits of animal research. Consideration may require researchers to facilitate and encourage those with the responsibilities for the care of animals to have the time, opportunities and confidence to develop systems which respect animals. It will also require merging with scientific knowledge supporting such ideals. For example the development of housing systems which align with the biological and psychological nature of the animals.

Respect for the essence of the animal may, furthermore, contribute to better science. For example, barren and confined environments, arguably indicative of one of our least respectful attitudes to animals, may compromise the animals and question the relevance of the results [26,76,77]. Part of caring is to respect the animal’s *telos*, the ratness of the rat (even though the predominant attitude to rats probably reflects the historical association between vermin, the plague and public health). *Telos* represents the interests and needs of an animal that matters to it by virtue of its biological and psychological nature [78]. The rat’s nature, for example, is that it is nocturnal, burrowing, social and a forager. How well do we allow experimental rats to express their *telos*? Clearly, enrichment programs increasingly being adopted, albeit not without consideration of their effectiveness and impact on the results, are an important part of respect for an animal’s *telos*. For example, designing a more functional environment spatially dissociating feeder and nest to induce a regular pattern of mother-offspring separation [76]. Similarly, limiting the availability of food, rather than providing it *ad libitum*, thereby reflecting the ecological uncertainty rats evolved in, may be optimal for long-term health [79,80,81] as well as the reliability and validity of the results of the research [26]—“good welfare probably is good science” [82].

Ethics, when understood as the systematic reflection of moral issues in the public sphere [83], invites or demands the involvement of those other than researchers and staff caring for animals. The costs and benefits of research are borne more widely than by just the animals and the researchers. Therefore a more engaging ethic could be developed not only by those responsible for animal care, but also by those with oversight of animal treatment (e.g., animal ethics committees, animal welfare inspectors) and those with an interest in animals (e.g., institutions advocating for responsible animal use, philosophers and ethicists, consumer representatives, and animal advocates). These groups have their own perspectives, roles and responsibilities for animal welfare and should have the confidence, resources and opportunities to fulfil those roles while being cognizant of the social, ecological and physical interdependence of humans and animals [84]. A more engaging or pragmatic ethic might help reconcile the tension drawn from balancing utility, societal constraints, and human attributes drawn from experiences, emotions and knowledge.

## 5. Discussion

It is suggested that there are opportunities to improve the predominant understanding of the justification of the use of animals in science, commonly an appeal to the benefits of using them. In addition, it appears convenient to disregard animal rights without acknowledging that they, along with many other initiatives, also aim to enhance animal welfare. Admittedly, and partly because of the extreme actions of some activists, animal rights is dismissed when a common understanding is that it refers to any and all ethical arguments calling for the fair treatment and protection of animals. Reflecting the middle ground position shared by many that the use of animals for human benefit is acceptable provided it is humane or based on caring, then it is suggested that this understanding is simplistic and inadequate.

A more sophisticated stance might, in addition to an appeal to the benefits of experimentation, acknowledge the more common understanding of animal rights and, in the spirit of the co-evolved human-animal relationship, give greater respect to the animal. A more true recognition of “animal rights” goes beyond meeting the requirements or needs of animals, underpinned by science and good practice, and extends to respect for the nature or *telos* of the animal. It is the spirit rather than the mechanics that matter, without going to the more revolutionary “subjects-of-a-life” animal rights position. This limited and minority view of rights may not be helpful, the more expansive view reflects a middle ground concern with our responsibilities to care for animals or provide for their needs. It is acknowledged though, that the term “rights” may be too entrenched, the singular minority understanding contributing to further polarized debate rather than illuminating understanding.

If we do not respect animals, but justify any suffering, are we not obliged to consider making research animals less sentient so they will suffer less [85]? While acknowledging such an approach might limit suffering, it seems counterintuitive to engineer a rat to be less than a rat, precisely because to do so does not respect the relationship between animal and human. Presumably for the same reasons, blind hens [18,86,87] would not be adopted as layers even if they had better welfare and were more productively efficient.

It is suggested that the reason the human-animal relationship in science is, in addition to historical [88] and contemporary excesses [89], so controversial or problematic, and so heavily scrutinized compared with other animal uses, is because the animal is not respected and gains little from the relationship, unlike a pastoral farm animal that enjoys adequate food and space *etc.*, even though its life is ended prematurely. Perhaps the real source of tension with animal experimentation, what makes it so troubling, is that the relationship is less than mutual or respectful; rats benefit little from confinement having lost their autonomy but not their dependency [90]. If the relationship is to be enhanced, then respect for the essence of the animal should have a more predominant place in animal ethics. Without such respect, our ethic is flawed and is open to charges of serving only to give a “cloak of respectability”, ensuring compliance with legislative requirements, legitimizing animal use rather than fully protecting animal rights, or even assuaging human guilt [27,91].

Ethics being the systematic reflection of moral issues in the public sphere [83], requires more open communication and transparency. A more sophisticated ethic drawing on the middle ground of human-animal relationships may require greater input from that middle ground. For example, if the benefits of animal experimentation accrue to wider society, it might be more relevant for society, rather than individual researchers, to explicitly give consideration to the claims of the benefits justifying the use of animals in science. Science could then assist with the dilemmas of the human-animal relationship, rather than being seen as a source of those dilemmas [54]. This is not to denigrate current systems of animal care largely reflective of past norms, but to consider how those norms could or might evolve. It is surely fair to at least signal the possibility of changing norms to those who make significant investments on society’s behalf.

Whilst acknowledging a place for extremes in moving the middle ground, be they philosophical animal rights or exclusive reliance on benefits, a more significant issue may be the factors influencing the middle ground. For much of human history, people have interacted directly with a range of animals for physical, emotional and spiritual sustenance and may have an innate attraction or love of life [92]. In contrast, many modern humans’ relationships with animals are increasingly being exclusively shaped by companion animals [93]. The special antipathy to the use of dogs in science illustrates this point. There is, furthermore, a view that modern science does not encourage us to see animals as fellow citizens of the biosphere—we are quietly disconnecting ourselves from the natural world [4]. It remains to be seen if the middle ground will continue to reflect the richness and diversity of the co-evolution of humans and animals.

## 6. Conclusions

To conclude, a more expansive understanding of ethics guiding animal research will need to draw on a plurality of values if it is to reflect the middle ground. Respect, which is apparent, is not captured by simple appeals to the benefits of experimentation. Indeed, other aspects are also apparent in forms as diverse as legislation and animal care, as they arguably are in farming [5] suggesting it may be relatively simple to develop a more expansive ethic for animals used in research. A more sophisticated understanding of ethics, and full acknowledgement of our experiences and virtues in caring for and respecting animals, is surely one of the aims of civilized people [94]. Can the spirit of a true relationship between human and animal evident in the thoroughbred horse (see Introduction) be extended to the laboratory rat? Currently, that relationship is, at best, reflected in the Gary Larsson cartoon of a mother rat admonishing her offspring “*Quit school?* Quit school? *You wanna end up like your father? A career lab rat?*”
